# Epitope Analysis of Anti-Myeloperoxidase Antibodies in Patients with ANCA-Associated Vasculitis

**DOI:** 10.1371/journal.pone.0060530

**Published:** 2013-04-05

**Authors:** Shen-Ju Gou, Peng-Cheng Xu, Min Chen, Ming-Hui Zhao

**Affiliations:** Renal Division, Department of Medicine, Peking University First Hospital, Peking University Institute of Nephrology, Key Laboratory of Renal Disease, Ministry of Health of China, Key Laboratory of Chronic Kidney Disease Prevention and Treatment (Peking University), Ministry of Education, Beijing, China; Keio University School of Medicine, Japan

## Abstract

**Objective:**

Increasing evidences have suggested the pathogenic role of anti-neutrophil cytoplasmic antibodies (ANCA) directing myeloperoxidase (MPO) in ANCA-associated vasculitis (AAV). The current study aimed to analyze the association between the linear epitopes of MPO-ANCA and clinicopathological features of patients with AAV.

**Methods:**

Six recombinant linear fragments, covering the whole length amino acid sequence of a single chain of MPO, were produced from *E.coli.* Sera from 77 patients with AAV were collected at presentation. 13 out of the 77 patients had co-existence of serum anti-GBM antibodies. Ten patients also had sequential sera during follow up. The epitope specificities were detected by enzyme-linked immunosorbent assay using the recombinant fragments as solid phase ligands.

**Results:**

Sera from 45 of the 77 (58.4%) patients with AAV showed a positive reaction to one or more linear fragments of the MPO chain. The Birmingham Vasculitis Activity Scores and the sera creatinine were significantly higher in patients with positive binding to the light chain fragment than that in patients without the binding. The epitopes recognized by MPO-ANCA from patients with co-existence of serum anti-GBM antibodies were mainly located in the N-terminus of the heavy chain. In 5 out of the 6 patients, whose sera in relapse recognize linear fragments, the reactivity to linear fragments in relapse was similar to that of initial onset.

**Conclusion:**

The epitope specificities of MPO-ANCA were associated with disease activity and some clinicopathological features in patients with ANCA-associated vasculitis.

## Introduction

Anti-neutrophil cytoplasmic antibody (ANCA)-associated vasculitis (AAV) comprises granulomatosis with polyangiitis (GPA), microscopic polyangiitis (MPA) and Churg-Strauss syndrome (CSS). ANCA are serological hallmarks for the above-mentioned small vessel vasculitis. Proteinase 3 (PR3) and myeloperoxidase (MPO) are two major target antigens of ANCA in AAV [1]. MPO is the most common target antigen of ANCA in Chinese patients with AAV [2]–[4]. Even in patients with a clinical picture of GPA, about 60% of them have ANCA directed to MPO [5], [6]. In addition, in about 4–14% of AAV patients, most often MPO-ANCA positive patients, have both serum ANCA and anti-glomerular basement membrane (GBM) antibodies [7], [8].

The pathogenic role of ANCA, especially MPO-ANCA, in AAV was confirmed by animal studies, *in vitro* studies and clinical observations [9]–[11]. It has been suggested by several studies that immunological characteristics of MPO-ANCA, including IgG subclasses, epitope specificity, the avidity and titre, were associated with the development of AAV [12]–[16]. Therefore, we speculated that the differences of the immunological characteristics of MPO-ANCA might contribute to the clinical and pathological heterogeneity.

In AAV, the level of MPO-ANCA was not always consistent with the disease activity [17]. Our previous study found that despite complete remission had been achieved; the avidity and titer of MPO-ANCA did not decrease significantly during remission, as compared to the active stage [18]. Therefore, it is reasonable to speculate that such inconsistency between ANCA levels and disease activity might be attributed to differences in epitope specificity of MPO-ANCA.

Our previous study has preliminarily suggested that the different conformational epitope recognition of MPO-ANCA might contribute to the different disease phenotypes (GPA or MPA) [19]. However, the difference in fine epitopes of MPO-ANCA from patients with different phenotypes needs further investigation. In addition, epitope mapping of MPO, especially linear epitopes, might also provide clues to the pathogenesis of MPO-ANCA-associated vasculitis.

In the present study, we produced six linear recombinant deletion mutants of MPO molecule and analyzed linear MPO epitopes using sera from AAV patients with and without co-existence of serum anti-GBM antibodies. The epitopes recognized by sequential sera of patients with AAV, who suffered at least one relapse, were also studied. The associations between the epitope specificities and clinico-pathological features of the patients were further analyzed.

## Materials and Methods

### Patients and Sera

Seventy-seven patients with AAV, diagnosed at Peking University First Hospital were recruited. All the patients met the criteria of the Chapel Hill Consensus Conference definition of AAV [20]. At the time of diagnosis, all the patients were positive for peri-nuclear ANCA (P-ANCA) and MPO-ANCA, and 13 out of the 77 patients had co-existence of serum anti-GBM antibodies. Among the 64 patients without serum anti-GBM antibodies, 21 were classified as GPA and the other 43 were classified as MPA. The diagnosis of GPA was established if both the following criteria were met: (i) Chapel Hill Consensus Conference (CHCC) definition [20], patients were classified as GPA if they had systemic vasculitis and the presence of granulomatous inflammation in a biopsy specimen of the respiratory tract or the presence of clinical signs strongly suggestive of granulomatous disease in the respiratory tract, which comprised involvement of the upper respiratory tract with nasal inflammation (purulent/bloody nasal discharge), sinusitis or otitis media or lower respiratory tract manifestion with pulmonary nodules, cavities or fixed infiltrate. (ii) American College of Rheumatology (ACR) classification criteria of GPA [21]. The diagnosis of MPA was based on the CHCC definition [20]. Patients were classified as MPA if they had systemic vasculitis, and the absence of granuloma formation in a biopsy specimen and the absence of clinical signs compatible with GPA, which is strongly suggestive of granulomatous disease.

The serum samples were collected at the time of diagnosis and before commence of immunosuppressive therapy. Patients with secondary vasculitis were excluded. Among the 64 patients without anti-GBM antibodies, ten patients showed sustained positivity in serum MPO-ANCA and experienced at least one relapse event during follow up. For each of these ten patients, three serum samples were collected respectively: (i) active phase of initial onset before initiation of immunosuppressive therapy; (ii) remission after immunosuppressive therapy; and (iii) active phase of relapse. “Remission” was defined as “absence of disease activity attributable to active disease qualified by the need for ongoing stable maintenance immunosuppressive therapy”, as described previously [22]. “Relapse” was defined as “recurrence or new onset of disease attributable to active vasculitis” [22].

Serum samples from 35 healthy blood donors were collected as normal control.

Sera from all subjects were obtained and kept at −70°C until use. The research was in compliance of the Declaration of Helsinki and approved by the ethics committee of the Peking University First Hospital. Written informed consent was obtained from each participant.

### Detection of MPO-ANCA and Anti-GBM Antibodies

ANCA assays were performed by indirect immunofluorescence (EUROIMMUN, Lübeck, Germany) using ethanol-fixed human neutrophils. Antigen-specific enzyme-linked immunosorbent assay (ELISA) was performed against purified myeloperoxidase (MPO) [23]. The sensitivity and specificity of the MPO-ANCA ELISA assay were 50% and 97%, respectively. Anti-GBM assays were performed by ELISA using purified bovine α(IV)NC1 as solid phase antigen, with confirmation of antibody specificity by ELISA against recombinant human α3(IV)NC1 [24].

### Preparation of Recombinant MPO Fragments

**Figure 1:**
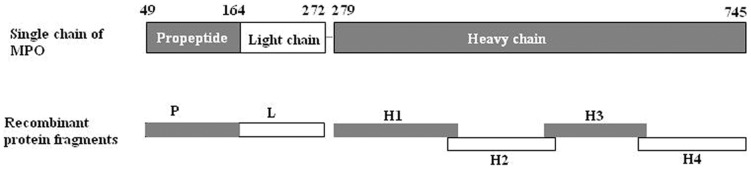
Schema of linear epitopes of MPO-ANCA. Six recombinant linear fragments, named P, L, H1, H2, H3 and H4, were produced using *E.coli*. P represents propeptide part, amino acids (aa) 49–164; L represents light chain, aa165–272; H1–H4 represent four fragments of the heavy chain, H1 for aa279–409, H2 for aa399–519, H3 for aa510–631 and H4 for 622–745. There were about 10 amino acids overlapping between the two adjacent fragments on the heavy chain.

Six linear fragments of myeloperoxidase (MPO), i.e., P, L, H1, H2, H3 and H4 were prepared as deletion mutants of MPO from *E.coli*. The amino acid sequences of the six fragments were as follows: 49–164 for propeptide (P), 165–272 for light chain (L), 279–409 for the N terminal of the heavy chain (H1), 399–519 for the second part of the heavy chain (H2); similarly, 510–631 for H3 and 622–745 for H4 (Figure 1). The cDNA of human MPO was a gift from Dr. Thomas Hellmark of Lund University Hospital.

### Synthesis of Primer Oligonucleotides

Primer oligonucleotides derived from the cDNA sequences of MPO were synthesized by Invitrogen Company. The sequences of them were as follows. 5′-primers: P, ATGGGGGTTCCCTTCTTCTCTTCTCTCAG; L, CGCGCGGCAGCCATATGGTGACTTGCCCGGAGCAGGAC; H1, GGGAATTCCATATGGTCAACTGCGAGACCAG; H2, TTCCTGGCAGGG GAC ACC CGT TCC; H3, GGGAATTCCATATGTTCCGCCTGGACAATCG; H4, AGG AAC CTG AAA TTG GCG AGG AAA CT. 3′-primers: P, CAT AAG CTT TCA TCC GAC GTC CTGGTAG; L, TGCTCGAGTGCGGCCGCTCACCGGGCGGCCGGCTCAGGGGTGAAG; H1, CCGCTCGAGCATCTCACTGGAACGGGTG; H2, CTT AAG CTT TTA CAT TGG CTG GTA CCG; H3, CCGCTCGAGCATCAGTTTCCTCGCCAAT; H4, CTC AAG CTT TTA CTA GGA GGC TTC CC.

### Amplification of DNA Fragments by Polymerase Chain Reaction (PCR)

MPO cDNA encoding each fragment was amplified by PCR. The reaction mixture (50 µl) contained 50 ng of the cDNA clone as a template, 1 µl of the upstream primer (10 µM), 1 µl of the downstream primer (10 µM), 25 µl of 2×Lamp MasterMix. The amplification was performed in a DNA thermal cycler by programmed incubation for 30 sec at 94°C, for 30 sec at 55°C, and for 30 sec at 72°C and repeated 30 times.

### Construction of MPO Fragments Genes

The PCR products of different MPO fragments were digested with enzymes and inserted into the plasmid vectors. L chain (L) were digested with enzymes NdeI and NotI and inserted into the expression vector pET28a (Merck, Germany). P, H2 and H4 were inserted into Hind III and PshA I sites of plasmid pET-42a(+) (Novagen, USA). H1 and H3 was inserted into NdeI and XhoI sites of plasmid pET-30a(+) (Novagen, USA).

### Confirmation of the Base Sequences of the Fragments

After the ligation of plasmid vectors and the target fragments, the newly formed plasmids were transformed into competent bacteria DH5a (Cwbio, Beijing, China). Positive bacteria were selected by selective culture-medium and the positive plasmid was sequenced by SinoGenoMax Company (Beijing, China). The base sequence of the target fragments were confirmed by DNAMAN software. The correctly constructed plasmid were selected and transformed into competent bacteria BL21 (Cwbio, Beijing, China) for expression.

### Expression of Recombinant P, L, H1, H2, H3 and H4 Constructs in Vectors

*E. coli* expressing each fragment of MPO was inoculated into 10 ml of Luria-Bertani medium (tryptone 10 g/L, yeast extract 5 g/L, NaCl 10 g/L) supplemented with kanamycin (100 µg/ml) in a shaker flask. The cells were grown with shaking (250 rpm) overnight at 37°C. The bacterial suspension was diluted to 1/100 with fresh medium and further incubated at 37°C with shaking at 250 rpm. When the absorbance at 600 nm became approximately 0.6–0.8, IPTG (0.4 mM) was added, and the mixture was incubated for a further 4 hr. The cells were harvested by centrifugation at 8000 rpm for 2 min.

### Purification of Insoluble Proteins with 6x His-tagged Protein Purification Kit

The harvested bacterial cells were lysed by ultrasonic and the precipitant was collected. The inclusion bodies in the precipitant were suspended in buffer 1 consisting of 50 mM Tris, 1 mM EDTA and 0.5% Triton (pH7.9). After centrifugation at 10000 rpm for 20 min at 4°C, newly formed precipitant was collected and resuspended in buffer 2 consisting of 50 mM Tris, 1 mM EDTA and 1.0%Triton (pH7.9). Similarly, buffer 3 (50 mM Tris, 1 mM EDTA and 1 M urea, pH7.9), buffer 4 (50 mM Tris, 1 mM EDTA, 2 M urea, pH7.9) and buffer 5(50 mM Tris, 1 mM EDTA, 4 M urea, pH7.9) were sequentially used to wash the insoluble target proteins in inclusion bodies. Then, the inclusion bodies were dissolved in 8 M urea buffer (50 mM Tris, 8 M urea, pH7.9) and the supernatant was collected by centrifugation at 10000 rpm for 20 min at 4°C. Finally, the target protein fragments were purified by 6x His-tagged protein purification kit (Cwbio, Beijing, China).

### Determination of the Purity of Recombinant Peptides

The recombinant proteins were treated with a sampling buffer (SDS 3 g, bromophenol blue 0.01 g, glycerol 2 ml, and 2-mecaptoethanol 1 ml, deionized water 15 ml) for 3 min at 100°C. Then the recombinant proteins were applied to 12% sodium dodecyl sulfate-polyacrylamide gel electrophoresis (SDS-PAGE). The gel was stained with MemGel staining solution (Applygen, Beijing, China).

### Determination of the Reactivity of Recombinant Proteins of MPO by ELISA

Highly purified recombinant MPO proteins were reconstituted to 10 µg/ml with coating buffer (0.05 M bicarbonate buffer, pH 9.6). A 100 µl portion of the mixture was then plated to a well of a polystyrene microtitre plate (Nunc Immunoplate; Nunc, Roskilde, Denmark) and kept overnight at 4°C. Every plate contained native MPO (2 µg/well) as a positive antigen control. The plate was washed three times with PBS containing 0.1% Tween-20 (PBST) (Chemical Reagents, Beijing, China). 2% BSA diluted by PBS was used to block the non-specific binding sites. The sera of subjects were diluted to 1∶100 by PBST/0.5 M NaCl (NaCl 0.5 M, KCl 2.7 mM, Na_2_HPO_4_ 10 mM, KH_2_PO_4_ 2 mM, pH 7.4) and were added in duplication. Every plate contained positive, negative and blank controls. The plate was incubated at 37°C for 1 hr and then washed three times with PBST, and the binding was detected with alkaline phosphatase-conjugated goat anti-human IgG (Fc specific; Sigma, St Louis, MO, USA) at a dilution of 1∶5000. The plate was washed three times with washing buffer and the P-nitrophenyl phosphate (pNPP, 1 mg/ml; Sigma) was used in substrate buffer [1 M diethanolamine and 0.5 mM MgCl_2_ (pH 9.8)]. The results were recorded as the net absorbance value (the absorbance values of peptide-coated wells minus the absorbance values of antigen-free wells) at 405 nm (A 405 nm) and samples were considered positive if the A 405 nm exceeded mean+2 S.D. (cutoff value) of the A 405 nm of the sera from 35 normal blood donors.

### Inhibition ELISA

Cross-reaction between antibodies against the recombinant proteins and native myeloperoxidase was investigated using inhibition ELISA. In brief, polystyrene microtiter plates were coated with myeloperoxidase. The diluted sera were preincubated with either myeloperoxidase or recombinants (P, L, H1, H2, H3 and H4), at increasing concentrations from 0.5 to 50 µg/ml at 37°C for 2 hr. The mixtures were then transferred to the myeloperoxidase-coated microtiter plates and the bound autoantibodies were detected with alkaline phosphatase-conjugated secondary antibodies, as described above. The absorbance value (OD) of the sera without the inhibitor was defined as 1, and the absorbance value of the sera preincubated with inhibitor was expressed relative to that. Similarly, the cross-reaction between antibodies against the H1 fragment and human α3(IV)NC1 was also performed using inhibition ELISA with a polystyrene microtiter plate coated with human α3(IV)NC1.

### Statistical Analysis

Differences in quantitative parameters were assessed using t test (for data that were normally distributed) or nonparametric test (for data that were not normally distributed). Differences in qualitative data were compared using χ^2^ tests. The difference was considered significant if a P value was <0.05. Analysis was performed with SPSS statistical software package (version 13.0, Chicago, IL, USA).

## Results

### General Data of Patients with AAV

Among the 64 AAV patients without anti-GBM antibodies, 32 were male and 32 were female, with an age of 60.5±15.1 (range 15–83) years at diagnosis. The clinical and histopathological data of the 64 patients were listed in Table 1. The clinical and laboratory data of the 13 AAV patients with co-existing anti-GBM antibodies were listed in Table 2. Among the ten patients with AAV who experienced at least one relapse, the Birmingham Vasculitis Activity Scores (BVAS) were 19.8±6.6 (range 13–36) at initial onset, zero in remission and 7.7±3.2 (range 3–12) in relapse, respectively.

**Table 1:** Clinical, laboratory features and results of AAV patients without anti-GBM antibodies.

Parameters	Number (%)
Number	64
Male/female	32/32
GPA/MPA	21/43
Age (years)	60.48±15.14
Scr (µmol/L)	
Mean±SD	339.11±237.71
Range	70–1007
Renal insufficiency at diagnosis	48 (75%)
Urinary protein (g/24 hr)	
Mean±SD	2.09±1.83
Range	0–7.25
ESR	70.05±40.14
Constitutional symptoms	49(76.6%)
Skin rash	7 (10.9%)
Arthralgia	15(23.4%)
Muscle pain	10 (15.6%)
Hemoptysis	10(15.6%)
Lung involved	43 (67.2%)
ENT	27(42.2%)
Ophthalmic	14(21.9%)
Gastrointestinal	11(17.2%)
Nervous system	10(15.6%)
BVAS	20.27±5.18
Glomeruli per biopsy	24.55±11.69
Percentage of normal glomeruli (%)	28.0±27.0
Glomerular lesions (%)	
Total crescents	60.58±27.19
Cellular crescents	28.42±25.99
Number of patients’ sera with positive reactivity to recombined linear peptides (GPA/MPA)	
P	4/5
L	3/8
H1	6/16
H2	6/11
H3	5/4
H4	5/8

[Abbreviations] GPA: granulomatosis with polyangiitis; MPA: microscopic polyangiitis; BVAS: Birmingham Vasculitis Activity Scores; ENT: ear, nose and throat; SD: standard deviation.

**Table 2:** Clinical and laboratory data of the AAV patients with co-existing anti-GBM antibodies.

Patient NO.	Sex	Age (years)	Initial Scr (µmol/L)	Anti-GBM level	MPO-ANCA level	Epitopes recognized
1	F	41	454	134	76	H1
2	M	13	500	87	137	H1
3	M	66	1000	116	68	H1
4	M	62	991	176	32	L/H1/H2/H3
5	M	66	1120	192	94	H1/H3
6	M	67	968	68	78	H1
7	M	64	291	40	83	−
8	M	70	1000	43	159	−
9	M	59	464	24	62	H1
10	F	77	1003	91	40	H1
11	M	67	165	64	95	−
12	F	45	740	83	176	−
13	M	58	218	85	75	−

‘−’, negative for any fragment test.

### Purification of Recombinant Proteins

**Figure 2:**
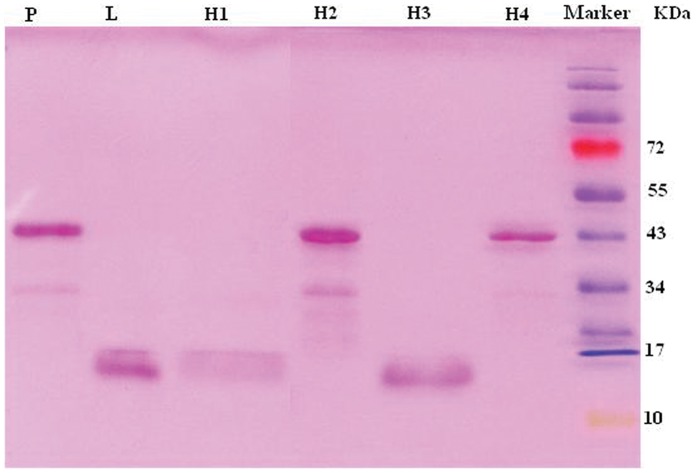
SDS-PAGE for recombinant protein fragments of MPO purified by the 6x His-tagged protein purification kit.

All the six recombinant MPO fragments were highly purified as proteins tagged with histidines with over 80% purity by Ni-NTA column chromatography. However, we failed to express the fragments P, H2 and H4 with hexamer histidine-tag. We could only express these three fragments with histidine and glutathione S-transferase (GST) tags simultaneously (Figure 2). No serum bound to GST tags, which was confirmed by both Western blot analysis and ELISA (data not show).

### The Reactivity of ANCA against the Linear Peptides of MPO

The cutoff values of positive reactivity, built up by normal sera testing, for recombinants P, L, H1, H2, H3 and H4 were 0.14, 0.14, 0.23, 0.20, 0.14 and 0.23, respectively.

The MPO-ANCA positive sera were demonstrated to recognize all the six constructed linear protein fragments. Sera from 45 out of the 77 (58.4%) patients could recognize at least one peptide.

**Figure 3:**
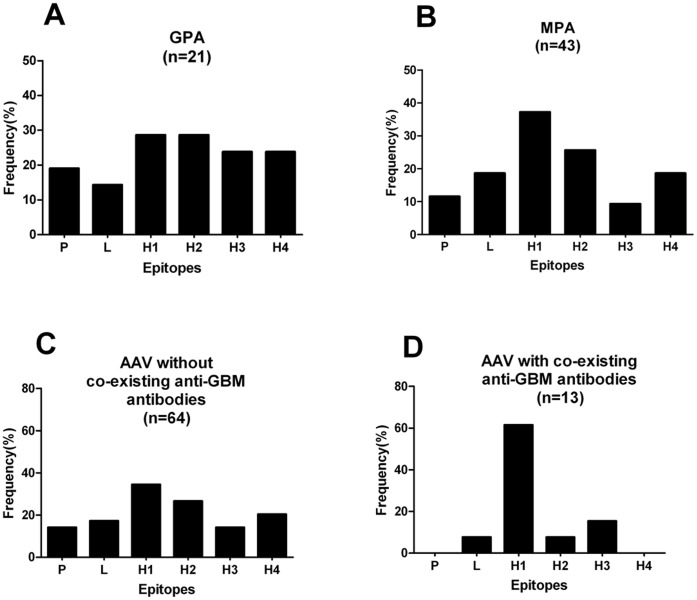
The frequencies of sera binding to different protein fragments in patients with different phenotypes. A. Patients with granulomatosis with polyangiitis (GPA). B. Patients with microscopic polyangiitis (MPA). C.P atients without co-existing anti-GBM antibodies. D. Patients with co-existing anti-GBM antibodies.

Among the sera from the 64 AAV patients without co-existing anti-GBM antibodies, 37(57.8%) showed a positive reaction to one or more linear fragments of the MPO chain. The reactivity distributed throughout the MPO molecule, although more frequently focused on the N-terminus of the heavy chain. No significant difference in the recognition frequency to different peptides was found between patients with GPA and patients with MPA. However, patients with GPA seemed to recognize the C-terminus of the MPO molecule (H3 and/or H4) more frequently than those with MPA (Figure 3A, 3B; table 1).

Among the 13 AAV patients with co-existence of anti-GBM antibodies, sera of 8(61.5%) patients recognized linear protein fragments of MPO. Interestingly, all these patients reacted with the H1 fragment (Table 2). The reactivity was much more restricted to the H1 fragment, which was highly different from that of AAV patients without anti-GBM antibodies (Figure 3C, 3D; table 1). The recognition frequency to H1 fragment tends to be higher in AAV patients with anti-GBM antibodies than that of AAV patients without anti-GBM antibodies (61.5% vs. 34.4%, χ^2^ = 3.352, P = 0.067). In addition, six out of the 13 AAV patients with coexisting anti-GBM antibodies recognized one peptide only, while the number of fragments recognized by AAV patients without anti-GBM antibodies varies widely.

**Figure 4:**
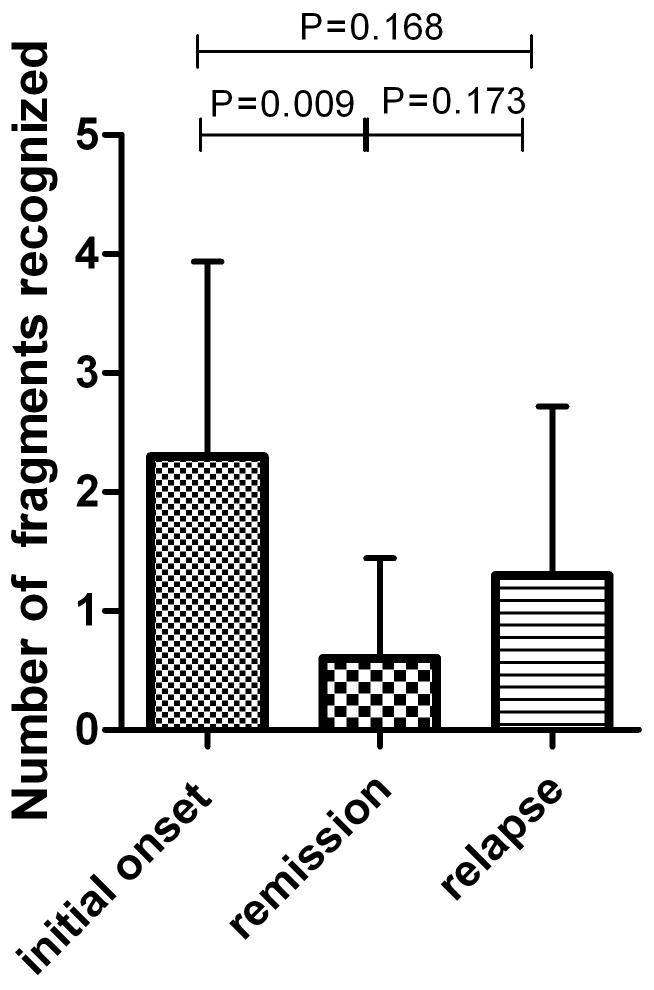
The number of protein fragments of MPO recognized in different stages of disease activity of AAV.

Among the 10 AAV patients who experienced relapse after remission, the number of fragments recognized in remission was significantly less than that in initial onset (2.0±0.6 vs. 5.0±2.3, P = 0.009, Figure 4). The binding pattern in 5 patients (patient No. 4, 5, 7, 8 and 10 in Table 3) changed obviously during a median follow-up of 30 (range 3–49) months. The binding to the linear peptides turned negative during follow-up in 4 of the 5 patients. The changing of the binding pattern in the other 5 patients was not obvious. In addition, in 5 out of the 6 patients, whose sera in relapse recognize linear fragments (patient No. 1, 2, 3, 6, 7 and 9 in Table 3), the reactivity to linear fragments in relapse was similar to that of initial onset. In addition, at initial onset of ANCA-associated vasculitis, 5 of 10 patients (patient No. 1, 6, 8, 9 and 10 in Table 3) recognized the H2 fragments. All of these binding to H2 fragments turned negative in remission (Table 3).

**Table 3:** Clinical, laboratory features and results of the ten patients with AAV who experienced relapse.

Patient NO.	Sex	Age (years)	Diagnosis	TI (months)	MPO-ANCA level			Peptides recognized		
					Initial onset	Remission	Relapse	Initial onset	Remission	Relapse
1	F	63	MPA	13	115	43	73	P/L/H1/H2/H4	−	P/H1
2	M	68	MPA	34	98	100	115	P/H1	H4	P/L/H1/H4
3	F	65	MPA	19	62	27	20	P	−	P/H1
4	F	70	GPA	3	105	29	33	H1	L	−
5	M	44	MPA	25	158	146	148	L	−	−
6	F	65	MPA	8	20	71	115	H1/H2	−	H1
7	F	50	MPA	30	184	45	38	−	−	H4
8	M	71	GPA	49	88	39	170	P/H1/H2/H4	H1/H4	−
9	M	46	MPA	13	143	102	200	P/H2/H4	P/H4	P/H2/H4
10	M	52	MPA	34	92	172	116	P/H1/H2/H4	−	−

TI, time interval between the initial onset and the relapse of disease;

‘−’, negative for any fragment test.

### Association of the Target Fragments of MPO with Clinicopathological Features of Patients with AAV

Among the 64 AAV patients without anti-GBM antibodies, no significant differences in clinical parameters were found between patients with antibodies binding to the recombinant fragments and those without any binding.

The BVAS and the initial serum creatinine were significantly higher in patients with positive binding to L fragment than that in patients with negative binding (24.0±6.0 vs. 19.2±6.0, P = 0.021; 472.2±310.3 µmol/L vs. 311.5±213.1 µmol/L, P = 0.04, respectively). Patients with positive binding to L fragment had significantly higher proportion of gastrointestinal system involvement than those with negative binding (45.5% vs. 11.3%, χ^2^ = 5.25, P = 0.022). In addition, patients with antibodies to the light chain reacted to higher number of the recombinant fragments than those without [1 (1, 3) vs. 0 (0, 2), P = 0.023]. The BVAS were significantly lower in patients with positive binding to H1 fragment than that in patients with negative binding (17.18±6.54 vs. 21.60±5.55, P = 0.006). In renal histology of 47 patients who received renal biopsy, the percentage of normal glomeruli in the renal specimens was significantly higher in patients with positive binding to H4 than that in patients with negative binding (42.0±30.4% vs. 22.9±23.3%, P = 0.025). The percentage of glomeruli with cellular crescents in the renal specimens was significantly lower in patients with positive binding to H4 than that in patients with negative binding (16.0±14.0% vs. 33.2±27.8%, P = 0.01).

### Cross-reaction of Antibodies

**Figure 5:**
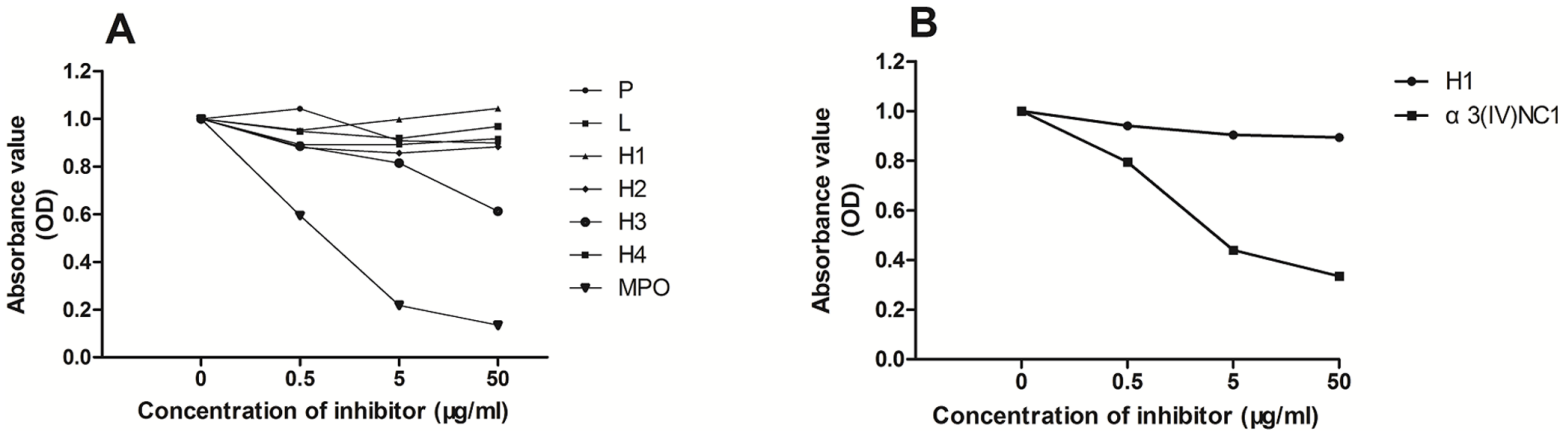
The results of antigen inhibition ELISA. Y axis: the absorbance value (OD) of sera bound with the antigen coated in the microtiter plates. The absorbance val ue (OD) of the sera without inhibitor was assigned a value of 1 and the OD of sera preincubated with inhibitor was expressed relative to that. X axis: different concentrations of the inhibitors for preincubation with the sera.

To investigate the cross-reaction between antibodies against the recombinant proteins and native myeloperoxidase, inhibition ELISA was performed. Binding of native myeloperoxidase IgG was strongly inhibited by soluble myeloperoxidase and H3 (inhibition rate >30%), but not the recombinant fragments P, L, H1, H2 or H4 (inhibition rate <30%, Figure 5A). This may indicated that antibodies against P, L, H1, H2 and H4 were distinct from that against native myeloperoxidase, while antibodies against H3 partially cross-reacted with that against native myeloperoxidase.

Since all the 8 patients with anti-GBM antibodies and anti-fragments antibodies reacted to the H1 fragment, amino acid sequences of α3(IV)NC1 and H1 fragment were compared using DNAMAN software. The integral identity of the two sequences was low (2.6%), but the amino acid sequences 107–112 (GRALEP) of α3(IV)NC1 were quite similar to amino acid sequences 373–378 (GRALLP) of MPO in H1 fragment. In addition, inhibition ELISA was also performed to investigate the antigenic similarity of α3(IV)NC1 and the H1 fragment. Binding of α3(IV)NC1 IgG was strongly inhibited by soluble α3(IV)NC1 but not the H1 fragment (Figure 5B), which indicated that antibodies against α3(IV)NC1 and H1 fragment were two distinct populations.

## Discussion

MPO-ANCA was confirmed to be pathogenic in ANCA-associated vasculitis by animal studies, *in vitro* studies and clinical studies [9]–[11]. Epitope specificity, one of the major immunological characteristics of MPO-ANCA, was speculated to be contribute to the heterogeneity of clinical features in AAV [14], [19]. To date, the epitope specificities, including both conformational and linear epitopes, of MPO-ANCA have not been clearly defined. Limited information is available with regard to whether the epitope specificities are associated with disease activities or clinicopathological features [25]–[28]. It has been demonstrated that MPO-ANCA mainly recognizes conformational epitopes on MPO molecule. However, linear epitopes of MPO might also provide important information on the pathogenesis of MPO-ANCA. The key amino acid motif or back bone amino acid structure derived from the fine linear epitope of MPO may also be used to identify potential causative microbial agents using molecular mimicry theory. To address these issues, we detected the epitope specificities of MPO-ANCA in sera of AAV patients with different phenotypes and patients at different stages of disease activities, i.e. initial onset, remission and relapse. Moreover, the associations between epitope specificities and clinicopathological features were also analyzed.

In the present study, we constructed six deletion mutants of the single chain of MPO molecule from the propeptide to the light chain and heavy chain. Propeptide is absent on mature MPO molecule, but it is a part of MPO precursor. Both mature MPO and the precursor form could be detected in the plasma [29]. We could not rule out the possibility that the propeptide might participate in breaking down the immune tolerance, so we constructed the propeptide as an epitope site. In the present study, 58.4% of sera from patients with MPO-ANCA positive vasculitis recognized one or more linear epitopes on MPO molecule. We speculated that the other patients might recognize conformational structures of MPO only. Among the 64 AAV patients without co-existing anti-GBM antibodies, 9 (14.1%) sera recognized the propeptide and 11 (17.2%) sera recognized the light chain. These were inconsistent with Suzuki’s study [28], [30], which reported the negative binding to the light chain. However, our results were consistent with the Bruner’s study [31], which demonstrated that the major epitope sites of MPO-ANCA existed on both the propeptide and the light chain. In Bruner’s study [31], the antigenic sequences identified on light chain included amino acids (aa) 213–222. In a recent study with a large sample size (74 patients with MPA) by Suzuki *et al* [28], the light chain was recombined in two parts divided by amino acid 214 and 215, which were among the aa 213–222 in Bruner’s study. The epitope sites on light chain might be just missed. In an early study by Suzuki’s group [30], the light chain was recombined as a whole, but only 4 patients were analyzed in the study. In the present study, the light chain was recombined as a whole, and the results suggested that the reactivity to light chain was not rare in Chinese patients with AAV. In addition, the binding to light chain of MPO-ANCA was associated with more severe renal dysfunction and systemic disease activity in AAV patients. Based on the three-dimensional model of MPO, the light chain is largely ‘hidden’ in the groove between the two MPO monomers and is poorly ‘accessible’ to antibody binding [32], [33]. Once the light chain was recognized, it might suggest that the epitope spreading was much more extensive, which may explain the severity in patients with MPO-ANCA reactivity to the light chain. In addition, patients with antibodies to the light chain reacted to higher number of the recombinant fragments than those without, which suggests an intra-molecular epitope spreading might happen.

In the current study, the epitope sites recognized by MPO-ANCA from AAV patients without serum anti-GBM antibodies displayed a more diverse repertoire, while the epitope sites recognized by MPO-ANCA from AAV patients with coexisting serum anti-GBM antibodies were much less diverse and mainly focused on the N-terminus of the heavy chain. The result indicated that the epitope specificities of MPO-ANCA were different between these two disease phenotypes, i.e. AAV patients with and without anti- GBM antibodies. It was also found in the current study that the recognition frequency to H1 fragment tends to be higher in AAV patients with anti-GBM antibodies than those without anti-GBM antibodies. In addition, the similarity of amino acid sequences 107–112 (GRALEP) of α3(IV)NC1 and amino acid sequences 373–378 (GRALLP) of MPO in H1 fragment raised the question that the H1 fragment might play a role in the development of the two antibodies (MPO-ANCA and anti-GBM antibodies), which still needs further investigation.

Whether the same epitopes are targeted during initial onset and relapse of AAV is an important issue and is not fully clear yet. In the present study, we tested sequential serum samples in 10 patients with AAV, at different stages of the disease, i.e., initial onset, remission and relapse. To the best of our knowledge, this is the first study to map the possible changing or epitope spreading of MPO-ANCA in AAV patients during the relapse of the disease. It was found that in the sera from relapse which recognized linear fragments, the reactivity to linear fragments was similar to that of initial onset. This may suggest a role of “immunological memory” for inducing relapse. In addition, although there was no fixed antibody-binding changing pattern in the three different stages of AAV, the number of recognized epitope sites tends to decrease while remission was achieved. Interestingly, the bindings to H2 fragment in active stage in 5 of the 10 patients all turned negative in remission. The association between binding to H2 fragment and disease activity raised the question that disease-inducing or risk epitope might exist within H2 fragment, which still needs further investigation.

In the present study, about 41.6% of the patients could not recognize the recombined linear peptides. We speculate that the epitopes recognized by these sera might be conformational only. However, those epitopes were not able to be detected with the method employed in this study.

In conclusion, this study provided evidence that MPO-ANCA could recognize linear epitopes throughout the corresponding antigen molecule MPO. The epitope specificities of MPO-ANCA were associated with disease activity and clinical and pathological manifestations in patients with ANCA -associated vasculitis.
